# Unveiling the Pharmacological Promise of *Rondeletia leucophylla*: A Multidimensional Approach

**DOI:** 10.1155/bmri/9565273

**Published:** 2025-11-14

**Authors:** Najmus Sakib Minhaj, Rajib Das, Sadia Afreen Chowdhury, Monira Ahsan

**Affiliations:** ^1^ Department of Pharmaceutical Chemistry, Faculty of Pharmacy, University of Dhaka, Dhaka, Bangladesh, du.ac.bd; ^2^ Department of Clinical Pharmacy and Pharmacology, Faculty of Pharmacy, University of Dhaka, Dhaka, Bangladesh, du.ac.bd; ^3^ Department of Pharmacy, School of Life Sciences, United International University, United City, Madani Avenue, Dhaka, Bangladesh, uiu.ac.bd; ^4^ Department of Pharmacy, Faculty of Sciences, Stamford University Bangladesh, Dhaka, Bangladesh, stamforduniversity.edu.bd

**Keywords:** antidiarrheal, anti-inflammatory, antimicrobial, antioxidant, *Rondeletia leucophylla*, thrombolysis

## Abstract

**Background:**

*Rondeletia leucophylla* has traditionally been used to treat various ailments, though scientific evidence is limited. This study is aimed at exploring its phytochemical profile through in vitro, in vivo, and in silico investigations.

**Materials and Methods:**

The dried coarse powder of *R. leucophylla* stem and leaves was extracted with methanol, then concentrated and dried using a rotary evaporator. The extract was subsequently evaluated through in vitro and in vivo pharmacological assays, preliminary phytochemical screening with standard reagents, gas chromatography–mass spectrometry (GC‐MS) analysis, and various in silico approaches.

**Results:**

Phytochemical screening of the methanolic extract of *R. leucophylla* (MERL) revealed the presence of steroids, carbohydrates, and glycosides, while GC‐MS identified 70 bioactive compounds. MERL showed a total phenolic content of 34.075 mg GAE/*μ*g. Its DPPH assay indicated strong antioxidant activity (IC_50_: 28.87 *μ*g/mL) compared to the standard butylated hydroxytoluene (BHT, IC_50_: 26.82 *μ*g/mL). The extract also exhibited moderate thrombolytic activity (39.42%) and antimicrobial effects against various bacterial and fungal strains. The anti‐inflammatory results showed that the 400 mg/kg dose stopped paw edema by 49.01%, which is close to aceclofenac′s 65.19% reduction. MERL showed strong antidiarrheal action, lowering the number of feces by 87.84% at 600 mg/kg, which was about the same as the standard drug (90.54%). The hypoglycemic effect depended on the dose; the 400 mg/kg dose lowered blood sugar levels significantly close to the control dose (*p* < 0.001). Molecular docking revealed strong binding affinities of selected compounds to key oxidative stress‐related targets, exceeding standard benchmarks, while ADMET profiling indicated favorable drug‐like properties and low toxicity.

**Conclusion:**

This study supports the traditional use of *R. leucophylla*, highlighting its antioxidant, thrombolytic, antimicrobial, antidiarrheal, anti‐inflammatory, and hypoglycemic potentials, warranting further pharmacological exploration.

## 1. Introduction

Medicinal plants have been a cornerstone of conventional medicine for ages, providing a prolific source of bioactive compounds utilized to treat a variety of diseases. The therapeutic potential of plants is largely attributed to their diverse phytochemical constituents, which exhibit several pharmacological actions, including antioxidant, antimicrobial, thrombolytic, anti‐inflammatory, and antidiarrheal properties. Among these medicinal plants, for centuries, sophisticated traditional medical systems have been founded on flora. The World Health Organization (WHO) says that traditional medicines are the main way that 80% of people around the world get basic medical care [1]. These natural preparations continue to be integral to both preventive and therapeutic medical strategies, contributing several key bioactive molecules that form the basis of modern pharmaceuticals. Globally, 119 compounds derived from plants are currently used in Western medicine, and 12 of the Top 25 bestselling drugs originate from natural sources [2]. The pharmacological properties of medicinal plants are largely due to bioactive secondary metabolites, including flavonoids, alkaloids, terpenoids, saponins, phenolics, essential oils, and tannins [3].

*Rondeletia leucophylla*, a member of the Rubiaceae family, has garnered attention for its ethnomedicinal applications, yet remains underexplored in scientific literature. It is a flowering plant native to tropical and subtropical regions. The Neotropics are inhabited by the genus *Rondeletia*, which belongs to the Rubiaceae family of flowering plants. Because of its small stature, *R. leucophylla* is ideally suited as a bonsai specimen. The family is the fourth biggest Angiosperm family with 13,143 species in 611 genera [4]. Because of their richness and pharmacological characteristics, many species in the Rubiaceae family have shown promise as sources for the creation of novel possible metabolites and drug prototypes [5]. Species within this family have demonstrated a broad spectrum of bioactivities, indicating their potential as sources of novel therapeutic agents.

Oxidative stress is a state marked by a lack of equilibrium between free radicals and antioxidants inside the body, leading to cellular damage and contributing to the emergence of long‐term conditions including diabetes, cancer, and heart disease [6]. Antioxidants are molecules that are capable of offering an electron to counteract the effects of free radicals, thereby preventing cellular damage. Natural antioxidants from plant sources are highly sought after due to their effectiveness and safety compared to synthetic antioxidants. Standard assays such as the 2,2‐diphenyl‐1‐picrylhydrazyl (DPPH) radical scavenging method and total phenolic content (TPC) analysis are employed to evaluate antioxidant capacity [7, 8]. Previous studies have shown that plants with high phenolic and flavonoid content exhibit significant antioxidant activity [9]. Given the traditional use of *R. leucophylla* in treating oxidative stress‐related conditions, it is imperative to investigate its antioxidant potential.

Infectious illnesses continue to pose a serious risk to world health, exacerbated by the emergence of drug‐resistant microbial strains. The need for new antimicrobial agents has intensified, and plant‐derived compounds offer promising alternatives [10]. Antimicrobial efficacy is commonly assessed using techniques such as the agar well diffusion method, which measures the inhibitory effect of extracts on microbial growth. Most phytochemicals have significant medicinal properties, including insecticidal, spasmolytic, antibacterial, antifungal, anticonstipation, antiplasmodial, and antioxidant properties, among others [11]. Investigating the antimicrobial properties of *R. leucophylla* can contribute to advancements in the creation of novel antimicrobial agents to combat resistant strains.

Thrombosis, the formation of a blood clot within a blood vessel, poses a significant risk for cardiovascular diseases, including heart attack and stroke [12]. Thrombolytic agents, or clot‐busters, are used to dissolve blood clots and restore normal blood flow. However, the current synthetic thrombolytic drugs have limitations, including adverse side effects and high costs. Thus, there is a growing interest in identifying natural thrombolytic agents from plant sources. Evaluating the thrombolytic potential of *R. leucophylla* could provide a safer and more affordable alternative to synthetic thrombolytic drugs. Similarly, inflammation, characterized by swelling, redness, and pain, is mediated by complex biochemical pathways [13]. While synthetic anti‐inflammatory agents are effective, they often come with adverse effects, driving interest in plant‐based alternatives [14].

Diabetes mellitus (DM), a chronic metabolic disorder, is another global health burden marked by hyperglycemia resulting from insulin insufficiency or resistance [15, 16]. Existing hypoglycemic agents, though effective, are associated with side effects such as hypoglycemia, obesity, and gastrointestinal issues [17]. Phytotherapy has shown efficacy in mitigating hyperglycemia, with over 800 plants identified as possessing significant antidiabetic properties [18]. Diarrhea is a fatal condition that impairs normal bowel function, characterized by increased stool water content, frequent defecation, and decreased absorption of fluids and electrolytes [15]. To treat diarrhea, oral rehydration solution, antisecretory, antimotility, and zinc supplements are typically used to reduce diarrhea drops and restore regular feces. Notwithstanding its effectiveness, the WHO has advocated for plant‐based therapies to address diarrheal episodes under its diarrhea control program [16].

Modern computational tools such as molecular docking have revolutionized drug discovery by allowing researchers to predict the interaction between plant‐derived compounds and biological targets. This cost‐effective and efficient approach helps identify lead compounds for further pharmacological development [19].

Given the traditional uses and the lack of scientific validation, the present study was designed to comprehensively evaluate the pharmacological activities of *R. leucophylla* using an integrated approach. Specifically, the methanolic extract was subjected to phytochemical profiling, antioxidant, antimicrobial, thrombolytic, anti‐inflammatory, antidiarrheal, and hypoglycemic assays, supported by gas chromatography–mass spectrometry (GC‐MS) analysis and in silico molecular docking. The overarching goal was to bridge traditional knowledge with experimental evidence and to provide a scientific rationale for the therapeutic potential of *R. leucophylla*.

## 2. Materials and Methods

### 2.1. Plant Material

*R. leucophylla* is mostly found in areas that are tropical or subtropical. In August 2022, a sample of *R. leucophylla* was gathered from the campus of Jahangirnagar University, located in Savar, Dhaka, Bangladesh. The botanical recognition of the plant was meticulously verified by specialists at the Bangladesh National Herbarium, located in Dhaka′s Mirpur. The plant was assigned the Accession Number JUH 10232, ensuring proper documentation and future reference. Following the collection and identification, the stems and leaves of the plant were subjected to a detailed preparation process to facilitate subsequent analyses. Initially, the plant material was thoroughly washed to remove any surface contaminants. After being washed, the plant was left to dry naturally in the shade for 11 days. This method was chosen to prevent the degradation of heat‐sensitive phytochemicals that might occur with direct sunlight or artificial drying methods. Once adequately dried, the plant material was crushed with a mechanical grinder, ensuring uniformity and consistency for the various tests to be conducted. The powdered plant material was then stored in airtight containers to protect it from moisture and environmental factors that could potentially alter its chemical composition. This preparation process was critical to preserving the integrity of the bioactive compounds present in *R. leucophylla*, thereby ensuring the reliability and accuracy of the subsequent phytochemical and pharmacological evaluations.

### 2.2. Chemicals and Reagents

The following chemicals were obtained from the respective suppliers: DPPH (Sigma Chemical Co.), Folin–Ciocalteu′s reagent (CDH Fine Chemical), Na_2_CO_3_ (Tradeasia International Pte. Ltd.), gallic acid (Anmol Chemicals), Mueller Hinton agar (MHA) (CDH Fine Chemical), streptokinase (SK) (Popular Pharma), ascorbic acid (SD Fine Chem. Ltd.), and loperamide and glibenclamide (Incepta Pharmaceuticals Ltd.).

### 2.3. Plant Extract Preparation

The collected *R. leucophylla* plant underwent a meticulous preparation process to ensure the integrity of its bioactive compounds for subsequent analysis. Initially, the dirt was carefully removed to extract the fresh stems and leaves. These parts were then thoroughly washed with room‐temperature water to eliminate all dust and impurities. Following the cleaning process, the plant materials were air‐dried in the shade for 12–15 days to prevent the degradation of heat‐sensitive phytochemicals. Once completely dried, the plant material was reduced to very small fragments and pulverized into tiny particles with a motorized mill to make sure of consistency. A total of 330 g of the powdered *R. leucophylla* were then subjected to maceration by being steeped in a sufficient amount of methanol to cover the powder by about three fingers′ depth. This mixture was periodically stirred over the course of 3 days to facilitate the extraction of phytochemicals. After the maceration period, the mixture was subjected to filtration with filter paper to differentiate the aqueous extract from the plant residue. The methanol solvent was allowed to evaporate naturally through air drying, resulting in a yield of 10.6 g of raw extract. This extract was carefully stored in a beaker, kept in a cool environment, and protected from direct sunlight to preserve its chemical integrity for further analysis and testing.

### 2.4. Experimental Animal

The study utilized Wistar rats (*Rattus norvegicus*) and Swiss albino mice (*Mus musculus*) of both sexes, ages 4–5 weeks, with corresponding weights of 140–150 g and 25–30 g. These animals were collected from Jahangirnagar University′s animal facility in Savar, Dhaka, Bangladesh. For 7 days, the animals were kept in polypropylene cages (dimensions: 30 × 20 × 13 cm) at the Institute of Food and Nutrition, University of Dhaka. Under standard laboratory conditions, including a temperature of 22^°^C ± 1^°^C, 12‐h light/dark cycles with relative humidity between 60% and 70%, they were strictly maintained. The animals received rodent water and food received from the International Centre for Diarrheal Disease Research, Bangladesh (ICDDRB). Understanding their sensitivity to environmental changes, the animals were introduced to the experimental setting for a minimum of 3–4 days before testing. Before the experiments started, the animals had a 12‐h fast during which they had access to only water. Ethical considerations were strictly followed throughout the study.

### 2.5. Phytochemical Screening

The analysis of phytochemicals was done by following the protocols that were outlined in the prior research [20].

### 2.6. Phytochemical Analysis Through the GC‐MS

*R. leucophylla* extract underwent phytochemical profiling utilizing a Shimadzu autosampler coupled with a GC‐MS‐QP2010 Ultra system (Shimadzu, Japan). The carrier gas was high‐purity helium, which flowed at a rate of 1.12 mL/min and had a linear velocity of 39 cm/s using a 5MS/HP column (30 m length, 0.25 mm internal diameter, and 0.25 *μ*m film thickness). Beginning at 110°C and running through a maximum of 280°C, the oven temperature was adjusted to rise at 10°C/min. The temperature was set to 250°C for the injection; a 50 *μ*L sample was then introduced splitlessly using a 10:1 split ratio. Whereas both the ion source and the MS transfer line were kept at 200°C and 250°C, respectively, the detector voltage was kept at 0.94 kV. Over a mass‐to‐charge (*m*/*z*) range of 85–500, full‐scan mass spectra were obtained at a scan rate of 10,000 *μ*/s. Matching the obtained spectra with the National Institute of Standards and Technology (NIST) library database helped to identify peaks and chemical ingredients.

### 2.7. Antioxidant Activity

The antioxidant activity of the methanolic extract of *R. leucophylla* (MERL) was assessed using the DPPH radical scavenging assay, a standard method to evaluate free radical quenching ability [21]. DPPH is a stable free radical with a deep violet color that absorbs at 517 nm; upon receiving an electron or hydrogen atom from antioxidants, it is reduced to a yellow‐colored DPPH. The degree of discoloration is proportional to the radical scavenging potential of the test compound. Briefly, different concentrations of MERL (0.977–500 *μ*g/mL) were prepared in methanol. One milliliter of each sample solution was mixed with 3 mL of a 0.1 mM DPPH solution in methanol and incubated for 30 min in the dark at room temperature to prevent photodegradation of radicals. After incubation, absorbance was recorded at 517 nm using a UV–vis spectrophotometer. Ascorbic acid and butylated hydroxytoluene (BHT) were used as standard antioxidants. Methanol served as the blank, while a DPPH solution without extract acted as the negative control. All measurements were performed in triplicate, and the percentage inhibition of DPPH radicals was calculated using the following formula:

%Inhibition=Absorbance of blank−Absorbance of test sampleAbsorbance of blank×100.

### 2.8. TPC

TPC was calculated utilizing a modified version of the Folin–Ciocalteu test [22]. Initially, incubation was performed on 40 *μ*L of the plant extract at ambient temperature for 5 min. Following this incubation, the extract was treated with 900 *μ*L of diluted Folin–Ciocalteu′s reagent. After thorough mixing, 400 *μ*L of a 15% Na_2_CO_3_ solution was incorporated, and the combination was permitted to react for 45 min at ambient temperature. Next, at 752 nm, the resultant solution′s UV absorbance was measured using a spectrophotometer. A blank solution, containing every reagent other than the plant extract, was used for baseline correction. To quantify the phenolic content, a standard curve was produced by the application of a series of gallic acid standard solutions. Milligrams of gallic acid equivalents per microgram of sample dried weight (microgram SD) was used to quantify the TPC of the extract. The reported results represent the mean of three separate tests, ensuring accuracy and reliability in the measurements.

### 2.9. Antimicrobial Test

#### 2.9.1. Test Microorganisms

The study was conducted on the four bacteria (Gram‐positive: *Bacillus cereus*, *Bacillus subtilis*, and *Streptococcus aureus* and Gram‐negative: *Escherichia coli*) and two fungi (*Saccharomyces cerevisiae* and *Aspergillus niger*). This antimicrobial susceptibility test was conducted in the microbiology laboratory at Stamford University, Bangladesh.

#### 2.9.2. Antimicrobial Susceptibility Test

Antimicrobial drugs are crucial in alleviating the worldwide burden of infectious illnesses [23]. A multitude of medicinal plants have been identified as significant sources of natural antimicrobial chemicals, serving as a possible option for the treatment of challenging bacterial infections [24]. In this study, the antimicrobial activity of the *R. leucophylla* extract was evaluated using the disc diffusion method. The disc diffusion procedure involved inoculating MHA plates with the test microorganisms. Sterilized paper discs (6 mm in diameter) were then placed on the agar surface [25]. The MERL was prepared by dissolving the extract in the appropriate solvents to achieve concentrations of 300, 500, and 700 *μ*g/mL. The petri dishes were kept at 4°C for 2 h to ensure the extract was evenly distributed within the agar. Following this initial incubation, a 24‐h incubation period was conducted on the plates at 37°C to cultivate microbes and interact with the extract. After the incubation period, the inhibitory zones of the discs were quantified and recorded. The diameter of the inhibitory zones was measured in centimeters to determine the antimicrobial activity. This method provided a clear indication of the effectiveness of the *R. leucophylla* extract in inhibiting the growth of the test microorganisms, highlighting its potential as a natural antimicrobial agent.

### 2.10. Thrombolytic Activity Assay

To assess the thrombolytic property of the *R. leucophylla* extract, the technique described by Miah et al. [26] was employed. Blood samples were taken from volunteers in good health and divided into sterile, preweighed vials, each holding 1 mL of blood. Subsequently, the vials were subjected to incubation at a temperature of 37°C for 45 min to allow the blood to coagulate and form clots. After the coagulation period, the generated serum was carefully removed from each vial, and the fresh weight of the vials containing the clots was recorded. This initial weight measurement provided the baseline for determining clot weight. Following this, each vial received 100 *μ*L of a plant extract solution with a concentration of 2 mg/mL. For comparison purposes, 30,000 I.U. of SK served as the positive control, representing a standard thrombolytic agent, and the negative control consisted of 100 *μ*L of distilled water, representing a nonthrombolytic condition. The vials then spent 90 min incubating at 37°C to facilitate the interaction between the plant extract and the blood clots. This incubation period allowed sufficient time for the extract to exert its potential thrombolytic effects. After the incubation, the fluid released from the clots was carefully drained, and the vials were weighed again to obtain the final clot weight.

The extent of clot lysis, or the thrombolytic activity, was quantified using the following formula:

%of clot lysis=AB×100.

Here, *A* and *B* stand for the weight of the discharged clot prior to and following therapy, respectively.

This formula calculates the percentage reduction in clot weight, providing a measure of the effectiveness of the plant extract in dissolving the clots. By comparing the clot lysis percentages induced by the plant extract, the SK control, and the water control, we can assess the relative thrombolytic potency of the *R. leucophylla* extract.

This methodical approach ensured a controlled environment for assessing the thrombolytic activity, allowing for accurate and reliable results. The findings from this evaluation could contribute to an understanding of the potential therapeutic uses of *R. leucophylla* in managing thrombotic conditions.

### 2.11. Anti‐Inflammatory Property

According to Azza and Oudghiri [27], the anti‐inflammatory effects of the MERL were assessed employing a model of paw edema caused by carrageenan. Rats′ right hind paw′s plantar surface was administered subcutaneously with 0.2 mL of 1% (*w*/*v*) carrageenan (Sigma‐Aldrich, St. Louis, United States) in saline using this approach in order to induce inflammation. A Plethysmometer was used to measure the paws′ sizes before and 4 h following the carrageenan shot (baseline) to figure out the level of edema. The following treatments were arbitrarily given to four groups of rats, each with six rats. The control group got orally normal saline (3 mL/kg body weight). The second group received an orally administered, generally used common anti‐inflammatory medication, aceclofenac (100 mg/kg body weight). Oral administration of the MERL at doses of 200 and 400 mg/kg body weight was given to the third and fourth groups correspondingly. Every therapy was scheduled 1 h before the carrageenan injection. Every group tracked the development of paw edema; reductions in paw diameter relative to the control group indicated the extract′s ability to reduce inflammation. This approach offers a consistent strategy for evaluating possible anti‐inflammatory drugs′ effectiveness. Every group of rats had existing paw edema computed with the following formula:

Mean paw edema at tth hour=Mean paw volume at tth hour−Mean paw volume at 0th hour.%inhibition of paw edema was computed by the following formula≔Vc−VtVc×100.

Here, *V*_*c*_ is the mean paw edema of the control group at time *t* and *V*_*t*_ is the mean paw edema at time *t* for the treatment group.

### 2.12. Antidiarrheal Activity

MERL was investigated for antidiarrheal properties following Sini et al.′s [28] method. Thirty mice were divided into five groups of six following an 18‐h fast. Every animal in every group received orally 0.4 mL of castor oil. The control group received 0.5% Tween 80 in distilled water 30 min after castor oil delivery. The second cohort was administered loperamide (3 mg/kg body weight), a standard medicine. The third, fourth, and fifth groups received MERL at doses of 200, 400, and 600 mg/kg body weight, correspondingly. Every animal was housed individually after administration. Four hours of hourly observation tracked the degree of diarrhea. On absorbent paper set on the transparent cage′s floor, defecation pellets were collected.

To determine the percentage of defecation inhibition, the following method was applied:

%Inhibition of defecation=Mean no.of defected pellets of control group−Mean no.of defected pellets of treated groupMean no.of defected pellets of control group×100.

### 2.13. Hypoglycemic Activity

Using a controlled experimental design, the hypoglycemic response to MERL in rats given glucose was evaluated. Following a 12‐h fast, 24 rats were divided at random into four groups, each including six rats. Group 1, the control, had 1 mL of 0.9% saline along with 1% Tween 80 and 1–2 DMSO drops. Given orally at 5 mg/kg body weight, glibenclamide served as the positive control for Group 2. Groups 3 and 4 (200 and 400 mg/kg bw, respectively) received MERL orally as a finely prepared suspension. Thirty minutes following the distribution of the test samples, each rat received an oral dosage of 1.5 mL of a 20% glucose solution at a rate of 2 g/kg body weight. For blood sample collecting, the tail veins were punctured using a sterilized needle. At 0, 30, 60, 90, and 120 min after glucose was given, as well as right before it was given, blood glucose levels were checked. A glucometer and reactive strips of glucose oxidase–peroxidase were used to monitor and record blood glucose levels at each interval, allowing for a comprehensive evaluation of hypoglycemic activity [29].

### 2.14. In Silico Molecular Docking Study of GC‐MS Identified Major Bioactive Compounds of Crude Extracts

An in silico molecular docking study was conducted on the major bioactive compounds identified through GC‐MS analysis of the crude extract. A chemical library was developed by retrieving compound information such as PubChem CIDs, molecular weights (MWs), molecular formulas, structures, and canonical SMILES from the PubChem database. Pharmacokinetic properties of the selected compounds were evaluated through ADMET profiling using SwissADME and ProTox 3.0 online tools. The 3D structures of ligands and target proteins were obtained from the PubChem and RCSB Protein Data Bank, respectively. Ligand structures were optimized using BIOVIA Discovery Studio by adding hydrogens and minimizing energy and then saved in PDB format. Protein structures were optimized by eliminating water molecules, heteroatoms, and cocrystallized ligands and maintaining the desired chain using BIOVIA Discovery Studio; further energy minimization was performed using Swiss‐PDB Viewer. Molecular docking was carried out using PyRx with AutoDock Vina, where docking parameters were set based on the active site of the receptor. The ligand with the lowest binding energy (in kilocalorie per mole) was selected for further analysis. The resulting protein–ligand complexes were visualized using PyMOL, and detailed interaction analysis, including bond types and distances, was performed using BIOVIA Discovery Studio Visualizer. Software tools employed in this study included BIOVIA Discovery Studio Visualizer 2024, PyRx, PyMOL, ChimeraX, Swiss‐PDB Viewer, and ChemDraw Professional 16.0.

## 3. Results and Discussion

### 3.1. Phytochemical Screening

A chemical group test was conducted on the leaves and stems of MERL to identify its phytochemical constituents. The results of this screening revealed the existence of glycosides, steroids, and carbohydrates. Conversely, the screening process showed that flavonoids, alkaloids, reducing sugars, saponins, and tannins were absent (Table 1).

**Table 1:** List of several chemical group tests conducted on *Rondeletia leucophylla*′s leaves and stems.

**Phytochemical constituent**	**Alkaloid**	**Flavonoid**	**Reducing sugar**	**Steroid**	**Saponin**	**Carbohydrate**	**Glycoside**	**Tannin**
MERL	−	−	−	+	−	+	+	−

*Note:* In this case, the symbol “+” denotes the phytochemical′ presence, whereas “−” denotes its absence.

The presence of steroids, glycosides, and carbohydrates suggests that the leaves and stems of MERL may possess significant biological activities. Steroids are known for their roles in various biological processes such as anti‐inflammatory and immunomodulatory effects. Glycosides are often associated with cardiac health and antimicrobial properties, while carbohydrates can have nutritional benefits and may play a role in cell signaling and energy provision. The absence of alkaloids, flavonoids, reducing sugars, saponins, and tannins narrows down the specific types of bioactive compounds that could be contributing to the observed pharmacological effects of MERL. These findings provide a foundational understanding of the phytochemical profile of *R. leucophylla*, indicating its potential for various therapeutic applications based on the presence of specific chemical groups. Further studies could delve into the specific mechanisms of action and the potential health benefits conferred by these compounds.

### 3.2. GC‐MS

**Figure 1:**
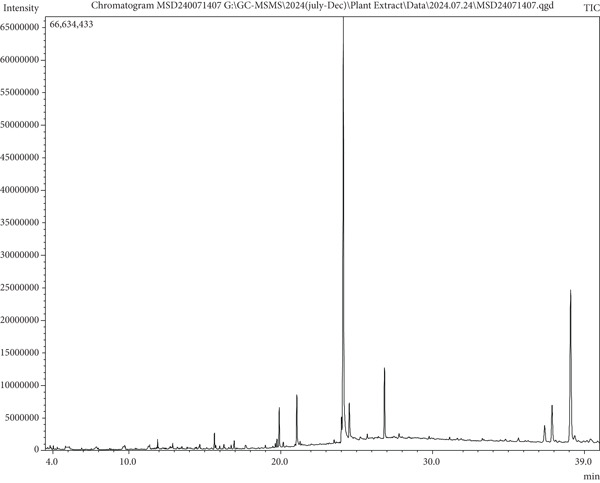
GC‐MS chromatogram of the MERL.

Table 2 features the active components found in the MERL together with information on their retention time (RT), chemical name, molecular formula, MW, and concentration stated as peak area percentage. Figure 1 shows the chromatographic results clearly, therefore exposing the existence of 51 bioactive phytochemical components in the extract. Emphasizing MERL′s capability as a source of physiologically active chemicals, this thorough investigation reveals its varied phytochemical profile.

**Table 2:** Phytocomponents identified in the MERL by GC‐MS.

**SL no.**	**Retention time**	**Peak area (%)**	**Compound name**	**Molecular formula**	**Molecular weight (g/mol)**
1	3.53	0.24	1,4‐Bis(trimethylsilyl)benzene	C_12_H_22_Si_2_	222.47
2	3.667	0.18	Benzeneethanamine, N‐trifluoroacetyl‐4‐hydroxy‐	C_10_H_10_F_3_NO_2_	233.19
3	3.826	0.36	3,3‐Dimethoxy‐2‐butanone	C_6_H_12_O_3_	132.16
4	3.894	0.09	1,3‐Dioxolane‐4‐methanol, 2‐ethyl‐	C_6_H_12_O_3_	132.16
5	5.896	0.09	1,1,3,3,5,5‐Hexamethyl‐1,5‐bis(2‐methylpropoxy)trisiloxane	C_14_H_36_O_2_Si_3_	320.70
6	8.617	0.19	Dodecane, 2,6,11‐trimethyl‐	C_15_H_32_	212.41
7	10.903	0.42	2,4‐Di‐tert‐butylphenol	C_14_H_22_O	206.32
8	11.225	0.1	Dodecane, 4‐methyl‐	C_13_H_28_	184.36
9	11.826	0.1	Dodecane, 2‐methyl‐	C_13_H_28_	184.36
10	12.203	0.15	Carbonic acid, eicosyl vinyl ester	C_23_H_44_O_3_	368.6
11	12.504	0.1	Eicosyl isopropyl ether	C_23_H_48_O	340.63
12	12.901	0.13	2‐Methyltetracosane	C_25_H_52_	352.68
13	13.377	0.13	Hexadecane	C_16_H_34_	226.44
14	13.615	0.16	Loliolide	C_11_H_16_O_3_	196.24
15	14.641	0.77	Neophytadiene	C_20_H_38_	278.52
16	14.724	0.27	2‐Pentadecanone, 6,10,14‐trimethyl‐	C_18_H_36_O	268.48
17	15.266	0.24	3,7,11,15‐Tetramethyl‐2‐hexadecen‐1‐ol	C_20_H_40_O	296.54
18	15.589	0.14	d‐Galactitol, 1‐O‐octyl‐	C_14_H_30_O_6_	294.39
19	15.939	0.45	Pentadecanoic acid, 14‐methyl‐, methyl ester	C_17_H_34_O_2_	270.45
20	16.735	0.12	Pimaric acid, TMS	C_20_H_32_O_2_Si	332.56
21	18.49	0.11	9‐Octadecenal, (Z)‐	C_18_H_34_O	266.46
22	18.653	0.23	9,12‐Octadecadienoic acid, methyl ester	C_19_H_34_O_2_	294.47
23	18.757	0.5	7‐Hexadecenoic acid, methyl ester, (Z)‐	C_17_H_32_O_2_	268.44
24	18.908	2.35	Phytol	C_20_H_40_O	296.54
25	19.178	0.3	Methyl stearate	C_19_H_38_O_2_	298.5
26	19.951	0.18	6‐Tert‐butyl‐2,3,4,9‐tetrahydro‐1H‐carbazol‐1‐one	C_16_H_19_NO	241.3
27	20.064	3.76	Hexadecanamide	C_16_H_33_NO	255.44
28	20.394	0.15	Dotriacontyl isopropyl ether	C_35_H_72_O	508.94
29	22.06	0.09	Methoxyacetic acid, 4‐hexadecyl ester	C_19_H_38_O_3_	314.5
30	22.899	0.09	4,8,12,16‐Tetramethylheptadecan‐4‐olide	C_21_H_40_O_2_	324.55
31	23.01	1.81	6,9‐Octadecadienoic acid, methyl ester	C_19_H_34_O_2_	294.47
32	23.133	39.52	9‐Octadecenamide, (Z)‐	C_18_H_35_NO	281.48
33	23.523	2.91	Tetradecanamide	C_14_H_29_NO	227.39
34	23.677	0.11	1,1,3,6‐Tetramethyl‐2‐(3,6,10,13,14‐pentamethyl‐3‐ethyl‐pentadecyl)cyclohexane	C_32_H_64_	448.86
35	23.844	0.14	11‐Methyltricosane	C_24_H_50_	338.66
36	24.251	0.19	(Z)‐14‐Tricosenyl formate	C_24_H_46_O_2_	366.62
37	24.925	0.15	Triacontane, 1‐bromo‐	C_30_H_61_Br	501.72
38	25.026	0.13	d‐Mannose‐methylhemiacetal, 1,2:3,4:5,6‐tri‐O‐(ethylboranediyl)‐	C_12_H_24_BO_6_	274.13
39	25.191	0.11	Octadecane, 3‐ethyl‐5‐(2‐ethylbutyl)‐	C_26_H_54_	366.71
40	25.295	0.18	Z‐3‐Methyl‐2‐hexenoic acid	C_7_H_12_O_2_	128.17
41	25.36	0.1	Octadecane, 1‐chloro‐	C_18_H_37_Cl	288.94
42	25.434	0.2	2‐Methylhexacosane	C_27_H_56_	380.73
43	25.54	0.13	(2E,4E)‐3,5,7‐Trimethylocta‐2,4‐dienedioic acid, O,O‐bis‐methyl	C_10_H_14_O_4_	198.21
44	25.843	5.01	Phthalic acid, di(2‐propylpentyl) ester	C_20_H_30_O_4_	334.45
45	25.99	0.1	1,1 ^′^:3 ^′^,1 ^″^‐Tercyclopentane, 2 ^′^‐dodecyl‐	C_17_H_32_	236.44
46	26.095	0.23	3,4‐Methylenedioxyphenyllactic acid, O,O ^′^‐bis(trimethylsilyl)‐	C_16_H_26_O_4_Si_2_	338.54
47	26.472	0.22	E,E,Z‐1,3,12‐Nonadecatriene‐5,14‐diol	C_19_H_34_O_2_	294.5
48	26.615	0.13	Nonadecyl heptafluorobutyrate	C_23_H_39_F_7_O_2_	480.55
49	27.005	0.12	Carbonic acid, octadecyl vinyl ester	C_21_H_40_O_3_	340.54
50	28.345	0.1	Decane, 5,6‐bis(2,2‐dimethylpropylidene)‐, (E,Z)‐	C_20_H_38_	278.53
51	28.93	0.21	Tetrapentacontane, 1,54‐dibromo‐	C_54_H_108_Br_2_	917.28
52	29.062	0.12	1,4‐Benzenedicarboxylic acid, bis(2‐ethylhexyl) ester	C_24_H_38_O_4_	390.56
53	30.127	0.19	Squalene	C_30_H_50_	410.72
54	30.888	0.12	Alpha.‐Tocospiro B	C_29_H_50_O_2_	430.71
55	31.474	0.1	Pentalene, octahydro‐2‐[(2‐octyl)decyl]‐	C_26_H_50_	362.7
56	32.285	0.16	3,21‐Bis(heptafluorobutyriloxy)‐3,5,10‐pregnatrien‐20‐one	C_29_H_26_F_14_O_5_	720.59
57	33.473	0.11	Gamma.‐Tocopherol	C_28_H_48_O_2_	416.68
58	33.813	0.17	Cholesta‐4,6‐dien‐3‐ol, (3.beta.)‐	C_27_H_44_O	384.64
59	34.66	0.33	Vitamin E	C_29_H_50_O_2_	430.71
60	35.296	0.12	9(11)‐Dehydroergosteryl benzoate	C_35_H_46_O_2_	498.77
61	36.383	2.09	Campesterol	C_28_H_48_O	400.68
62	36.875	4.28	Stigmasterol	C_29_H_48_O	412.69
63	37.118	0.27	Uvidin C, diacetate	C_31_H_50_O_4_	486.72
64	37.348	0.13	Ergosta‐5,22‐dien‐3‐ol, (3.beta.,22E)‐	C_28_H_46_O	398.66
65	38.097	18.99	Gamma.‐Sitosterol	C_29_H_50_O	414.71
66	38.367	1.17	Stigmastanol	C_29_H_52_O	416.72
67	38.619	0.26	9,19‐Cycloergost‐24(28)‐en‐3‐ol, 4,14‐dimethyl‐, acetate, (3.beta.,4.alpha.,5.alpha.)‐	C_32_H_52_O_2_	468.75
68	38.945	0.21	4‐Campestene‐3‐one	C_28_H_46_O	398.66
69	39.177	0.27	9,19‐Cyclolanostan‐3‐ol, acetate, (3.beta.)‐	C_32_H_52_O_2_	468.75
70	39.402	0.79	Lupeol	C_30_H_50_O	426.72

*Note:* Several entries represent derivatized forms (TMS derivatives) or common siloxane/phthalate contaminants. These were identified by library match and/or presence in reagent/solvent blanks and are reported as tentative; confirmation by LC‐MS/MS or NMR is required for unambiguous identification.

Figure 1 depicts the GC‐MS chromatogram of the MERL, revealing the complexity of its phytochemical composition. The chromatogram displays multiple well‐resolved peaks corresponding to a wide array of bioactive metabolites, as summarized in Table 2. Prominent constituents include phytol, stigmasterol, *γ*‐sitosterol, campesterol, lupeol, and several fatty acid esters, many of which are known for antioxidant, antimicrobial, and anti‐inflammatory activities. The presence of such structurally diverse metabolites highlights the chemical richness of MERL and provides a plausible explanation for its broad‐spectrum pharmacological effects observed in subsequent assays. These findings suggest that *R. leucophylla* could serve as a valuable reservoir of natural therapeutic agents and warrant further isolation and characterization of the individual compounds.

### 3.3. Antioxidant Activity

The extent to which DPPH radicals were suppressed was determined using ascorbic acid as the standard. Ascorbic acid exhibited 50% inhibition of the DPPH radical at a 26.82 *μ*g/mL concentration. In comparison, at 28.87 *μ*g/mL, the MERL was able to achieve 50% inhibition (Figure 2). This indicates that MERL has significant antioxidant activity, closely comparable to that of ascorbic acid. Free radicals and antioxidants can have both beneficial and detrimental effects on the body. Free radicals are generated internally through normal cellular metabolism and externally through exposure to radiation, tobacco smoke, environmental hazards, and certain medications. When free radicals accumulate in large numbers and cannot be neutralized, oxidative stress arises. Numerous illnesses′ etiologies are linked to oxidative stress, including autoimmune disorders, cancer, aging, cardiovascular diseases, and neurological disorders. The body possesses several intrinsic and extrinsic mechanisms to combat oxidative stress from environmental sources [30].

Figure 2IC_50_ value of (a) ascorbic acid and (b) MERL.

**Figure:**
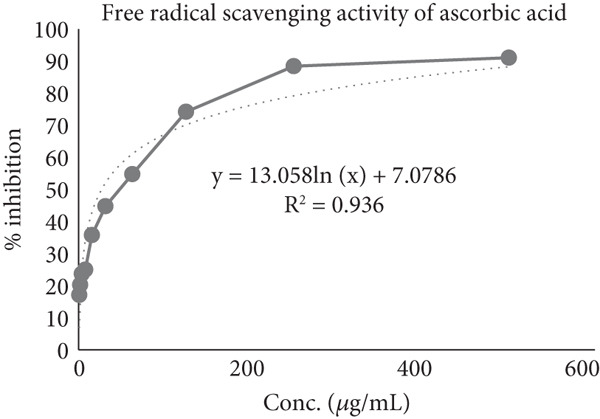
(a)

**Figure:**
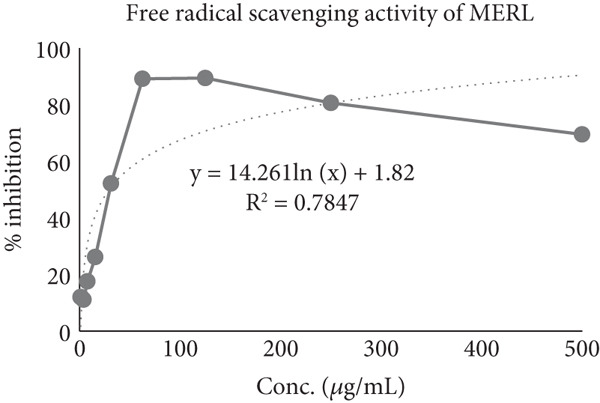
(b)

Scavenging oxygen radicals depend much on antioxidants including phenolic compounds and flavonoids. Phenolic compounds′ potential to act as hydrogen donors, singlet oxygen quenchers, and reducing agents is largely dependent on their redox characteristics, which also determine their antioxidant activity. The DPPH test demonstrated that the MERL has substantial antioxidant activity, as indicated by its capacity to suppress the DPPH free radical. The plant extract exhibited an antioxidant capacity equivalent to 28.87 *μ*mol Vitamin C per microgram, highlighting its potential in mitigating oxidative stress and associated health risks. These findings suggest that *R. leucophylla* has significant antioxidant properties that could be beneficial in preventing or managing diseases linked to oxidative stress. Further research could explore the specific mechanisms and potential health applications of this plant extract.

### 3.4. TPCs

**Figure 3:**
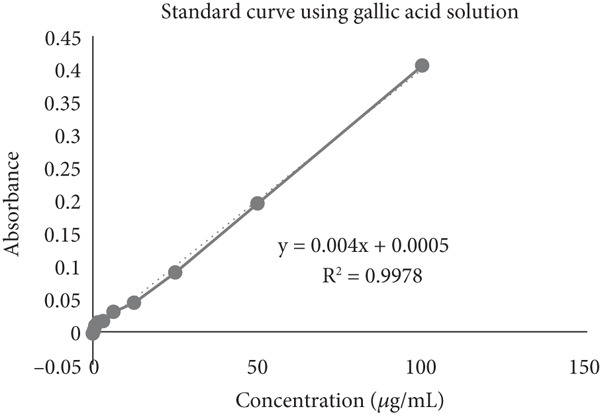
Gallic acid standard curve for total phenolic content analysis.

TPC analysis is pivotal in quantifying the amount of phenolic compounds present in plant samples [31, 32]. In this investigation, the Folin–Ciocalteu method was utilized to assess the TPC of MERL, as detailed in Table 3. This method involved calibration against a series of known gallic acid standards to find the amount of phenolics (Figure 3). The TPC of MERL was determined to be 34.075 mg GAE/*μ*g of sample in the methanol extraction tests. This finding indicates that the MERL contains a moderate concentration of polyphenolic compounds. Phenolic compounds are noteworthy because of their recognized antioxidant qualities, which include breaking free radical chains and acting as terminators of free radicals. These attributes are believed to contribute directly to the antioxidative capacity of the plant extract. Understanding the TPC of MERL provides insights into its potential health benefits, particularly in combating oxidative stress‐related diseases. Further exploration of these phenolic compounds could elucidate their specific mechanisms of action and medicinal uses, underscoring the significance of *R. leucophylla* as a potential source of antioxidants that are naturally occurring.

**Table 3:** Determination of TPC of MERL.

**Plant part**	**Sample code**	**Absorbance**	**Average**	**TPC**
Stem and leaves of *Rondeletia leucophylla*	MERL	0.142	0.137	34.075
		0.129		
		0.140		

### 3.5. Thrombolytic Activity

**Figure 4:**
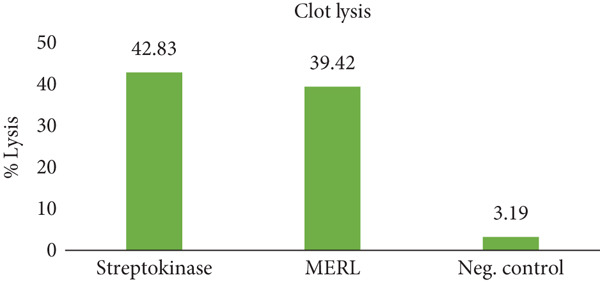
Mean value of percent of clot lysis.

The thrombolysis test is a controlled laboratory procedure utilized to evaluate the capability of the plant extracts to disintegrate blood clots. Blood and proteins bind together to form a clot when the body is wounded, limiting more bleeding. Thrombus development within blood vessels hinders blood flow via the circulatory system, causing anoxia, hypertension, heart attack, and so on [33]. The thrombolytic activity of the extracts of *R. leucophylla* was assessed as part of the investigation of medicines with a cardioprotective effect that are naturally occurring. The clots were treated with 100 *μ*L of SK, a positive control, which was subsequently kept at 37°C for 90 min, which broke up the clot. In contrast, the negative control, distilled water, demonstrated a minimal percentage of clot lysis (3.19%). A statistically significant variation in thrombus lysis percentage was found among both positive and negative controls on average. Figure 4 illustrates the thrombolytic activity of MERL compared with SK (positive control) and distilled water (negative control). The extract produced a mean clot lysis of 39.42%, which, while lower than SK (42.83%), was significantly greater than the negative control (3.19%). This moderate but notable thrombolytic potential indicates that MERL contains compounds capable of facilitating fibrinolysis or interfering with clot stability. Given that thrombotic disorders such as myocardial infarction and stroke are major contributors to global morbidity and mortality, the ability of MERL to promote clot breakdown suggests potential therapeutic relevance. Although the activity did not reach that of the standard drug, its natural origin, lower risk of adverse effects, and phytochemical complexity underscore its value as a lead source for the development of safer, plant‐derived thrombolytic agents.

### 3.6. Antimicrobial Activity

To evaluate the potency of antimicrobial agents, antibacterial and antifungal assays are necessary. Antibacterial tests are performed to determine the ability of a substance to inhibit the growth of bacteria, whereas antifungal tests are conducted to evaluate the potential of a substance to suppress the growth of fungi [32]. The antibacterial and antifungal characteristics of various concentrations of plant extract were investigated against Gram‐positive and Gram‐negative bacteria along with two distinct species of fungus by determining the zone of growth inhibition in millimeters. The findings demonstrated that the inhibitory zone expanded as plant extract concentration rose (Tables 4 and 5). The antibacterial activity inhibition zone was 07–15 mm, whereas the fungal inhibition zone measured 09–15 mm. The subsequent findings indicate that MERL has the strongest antibacterial action against *E. coli* and *B. cereus*, the pathogens responsible for serious diseases such as respiratory disorders, pneumonia, diarrhea, and gastrointestinal tract infections.

**Table 4:** Inhibition zone of MERL against different bacteria.

**Test organisms**	**The zone of inhibition′s diameter (mm)**			
	**MERL (300 *μ*g/disc)**	**MERL (500 *μ*g/disc)**	**MERL (700 *μ*g/disc)**	**Ciprofloxacin**
Gram‐positive				
*Bacillus cereus*	8	12	15	26
*Bacillus subtilis*	7	9	12	26
*Staphylococcus aureus*	8	10	13	27
Gram‐negative				
*Escherichia coli*	9	10	15	24

**Table 5:** MERL′s inhibition zone against fungi.

**Test organisms**	**The zone of inhibition′s diameter (mm)**			
	**MERL (50 *μ*g/disc)**	**MERL (500 *μ*g/disc)**	**MERL (700 *μ*g/disc)**	**Griseofulvin (50 *μ*g/disc)**
*Saccharomyces cerevisiae*	7	8	13	21
*Aspergillus niger*	8	9	10	20

### 3.7. Anti‐Inflammatory Activity

The plant extracts′ anti‐inflammatory qualities were evaluated by quantifying the percentage inhibition of paw edema in rats, generated by the test samples, and juxtaposing the findings with the standard medication aceclofenac. Aceclofenac demonstrated considerable suppression of paw edema, with values of 29.63%, 45.11%, 56.59%, and 65.19% at the 1st, 2nd, 3rd, and 4th hours, respectively, after medication taken orally (Table 6).

**Table 6:** Assessment of paw volume measurements in rats at various time intervals.

**Group**	**M** **e** **a** **n** **p** **a** **w** **v** **o** **l** **u** **m** **e** (**m** **L**) ± **S** **E** **M**			
	**1st hour**	**2nd hour**	**3rd hour**	**4th hour**
Control	0.72 ± 0.03464	0.7833 ± 0.0318	0.86 ± 0.03464	0.91 ± 0.01732
Standard	0.5067 ± 0.01856^∗∗∗^	0.43 ± 0.01528^∗∗∗^	0.3733 ± 0.01453^∗∗∗^	0.3167 ± 0.01333^∗∗∗^
MERL (200 mg/kg)	0.653 ± 0.024^∗∗∗^	0.615 ± 0.0212^∗∗∗^	0.5392 ± 0.01367^∗∗∗^	0.4825 ± 0.0216^∗∗∗^
MERL (400 mg/kg)	0.594 ± 0.0154^∗∗∗^	0.557 ± 0.0215^∗∗∗^	0.4912 ± 0.0116^∗∗∗^	0.464 ± 0.0171^∗∗∗^

*Note:* ^∗∗∗^ *p* < 0.001 compared with control (one‐way ANOVA followed by Dunnett′s test).

**Figure 5:**
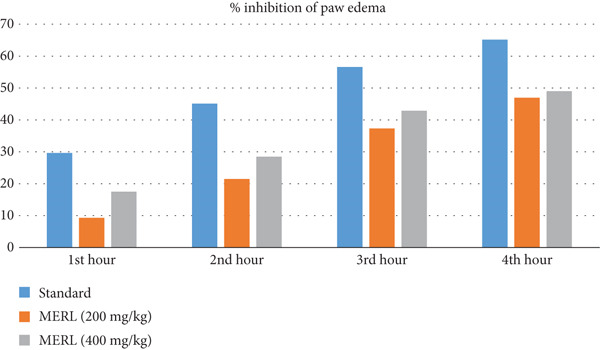
Comparative analysis of percent inhibition of edema across various groups.

MERL shows dose‐dependent anti‐inflammatory effects at 200 and 400 mg/kg. MERL at 200 mg/kg inhibited paw edema from 9.31% to 46.98% in the first 4 h, whereas at 400 mg/kg, it demonstrated 17.5%–49.01% inhibition during the same timeframe (Figure 5). Research indicates that MERL has significant anti‐inflammatory properties, equivalent to aceclofenac.

### 3.8. Antidiarrheal Activity

**Figure 6:**
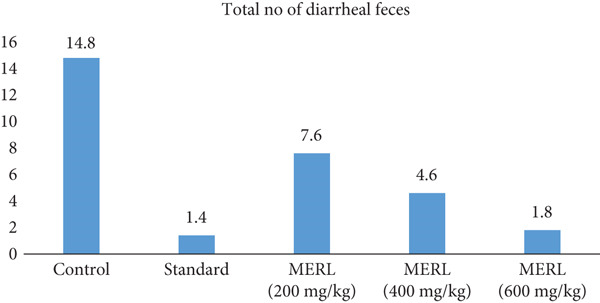
An analysis of the standards′ and MERL test compounds′ total diarrhea fecal counts.

MERL was tested for antidiarrheal action at dosages of 200, 400, and 600 mg/kg, in contrast with a control group and a standard medication group (25 mg/kg) (Figure 6). In the control group, mice generated an average of 14.8 ± 1.28 diarrheal feces, with no signs of inhibition. Defecation was greatly decreased by the usual treatment, with an average of 1.4 ± 0.40 diarrheal feces and a 90.54% inhibition rate (*p* < 0.001) in contrast with the control. MERL at 200 mg/kg reduced diarrheal feces to an average of 7.6 ± 0.68, resulting in a 48.65% defecation inhibition rate (*p* < 0.001). At 400 mg/kg, diarrheal feces decreased to an average of 4.6 ± 0.51, indicating a 68.92% inhibition rate (*p* < 0.001). The highest dosage of 600 mg/kg had the greatest impact, resulting in an average of 1.8 ± 0.37 diarrheal feces and an 87.84% inhibition rate (*p* < 0.001). These results imply that MERL has dose‐dependent antidiarrheal action. The control, standard, and test materials affected the feces patterns of research mice.

The 600 mg/kg MERL dosage had an antidiarrheal effect equivalent to the standard medication. This shows that MERL may be useful for treating diarrhea. Additional study is necessary to determine and understand the active chemicals responsible for its antidiarrheal effects and their processes.

### 3.9. Hypoglycemic Activity

**Figure 7:**
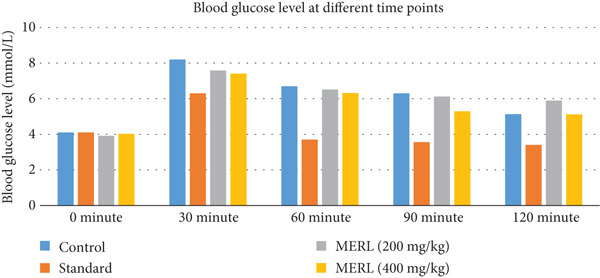
Comparison of rats′ blood level of glucose (millimoles per liter) at certain periods.

MERL was tested for hypoglycemic activity at 200 and 400 mg/kg dosages, comparing findings to control and standard medication groups. Blood glucose was tested at 0, 30, 60, 90, and 120 min. The control group experienced a considerable rise in blood glucose levels, peaking at 8.2 ± 0.321 mmol/L after 30 min and subsequently decreasing to 5.13 ± 0.441 at 120 min. The conventional medication group showed substantial hypoglycemia effects throughout observation. Blood glucose levels decreased significantly from baseline (4.1 ± 0.252 mmol/L) to 6.63 ± 0.384 at 30 min and 3.4 ± 0.173 at 120 min (*p* < 0.001 for all time periods compared to control). MERL 200 mg/kg has mild hypoglycemic action. Compared to the control, blood glucose levels climbed to 7.58 ± 0.12 mmol/L at 30 min, then steadily declined to 6.51 ± 0.22 at 60 min (*p* < 0.01) and 5.89 ± 0.26 at 120 min (*p* < 0.01). MERL 400 mg/kg hypoglycemia impact was stronger. In comparison to the control group, blood glucose levels reached a maximum of 7.41 ± 0.31 mmol/L at 30 min (*p* < 0.001) and fell to 6.32 ± 0.1 at 60 min and 5.12 ± 0.11 at 120 min (Table 7 and Figure 7).

**Table 7:** Comparative hypoglycemic action among various test material groups, standard, and control.

**Sample code**	**M** **e** **a** **n** **b** **l** **o** **o** **d** **g** **l** **u** **c** **o** **s** **e** **c** **o** **n** **c** **e** **n** **t** **r** **a** **t** **i** **o** **n** (**m** **m** **o** **l**/**L**) ± **S** **E** **M**				
	**0 min**	**30 min**	**60 min**	**90 min**	**120 min**
Control	4.1 ± 0.11	8.2 ± 0.32	6.7 ± 0.15	6.3 ± 0.15	5.13 ± 0.44
Standard	4.1 ± 0.25	6.3 ± 0.38^∗∗∗^	3.7 ± 0.2^∗∗∗^	3.56 ± 0.22^∗∗∗^	3.4 ± 0.17^∗∗∗^
MERL (200 mg/kg)	3.91 ± 0.3	7.58 ± 0.12^∗^	6.51 ± 0.22^∗∗^	6.12 ± 0.17	5.89 ± 0.26^∗∗^
MERL (400 mg/kg)	4.02 ± 0.21	7.41 ± 0.31^∗∗∗^	6.32 ± 0.1^∗∗∗^	5.29 ± 0.15^∗∗∗^	5.12 ± 0.11^∗∗∗^

*Note:* ^∗∗∗^ *p* < 0.001,  ^∗∗^ *p* < 0.01, and  ^∗^ *p* < 0.05 compared with control (one‐way ANOVA followed by Dunnett′s test).

MERL has a dose‐dependent hypoglycemic effect, according to the study. The 400 mg/kg dosage was more efficacious than the 200 mg/kg dose, with effects similar to the usual medication. MERL may be a promising natural hypoglycemic drug, requiring additional research into its active components and methods of action.

### 3.10. Molecular Docking Study to Evaluate Antioxidant Activities of GC‐MS Identified Major Compounds of MERL

**Figure 8:**
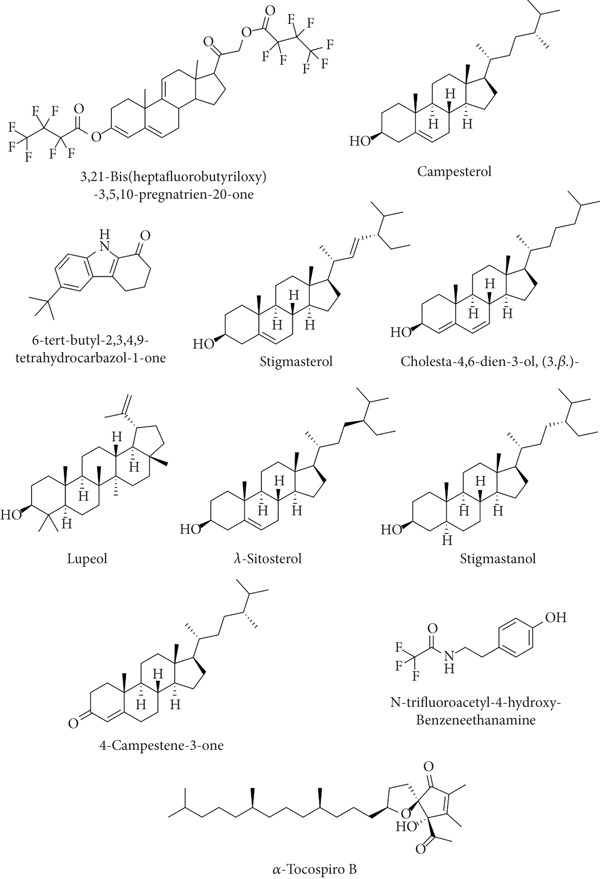
Molecular structure of selected ligands of MERL.

Eleven of the 70 significant GC‐MS compounds were docked with CBR‐1 protein (PDB ID: 4Z3D). These compounds met all ADMET requirements and were drug‐like. Therefore, they were chosen for molecular docking analysis. The selection was based on Lipinski′s rule of five parameters such as MW, number of hydrogen bond donors and number of hydrogen bond acceptors, lipophilicity (expressed as LogP), and water solubility. ADMET analysis of these selected ligands is as follows:

Beta carotene was the antioxidant standard. From PubChem, 3D structures of chosen bioactive compounds were extracted, and Figure 8 shows the molecular structures of selected ligands.

**Figure 9:**
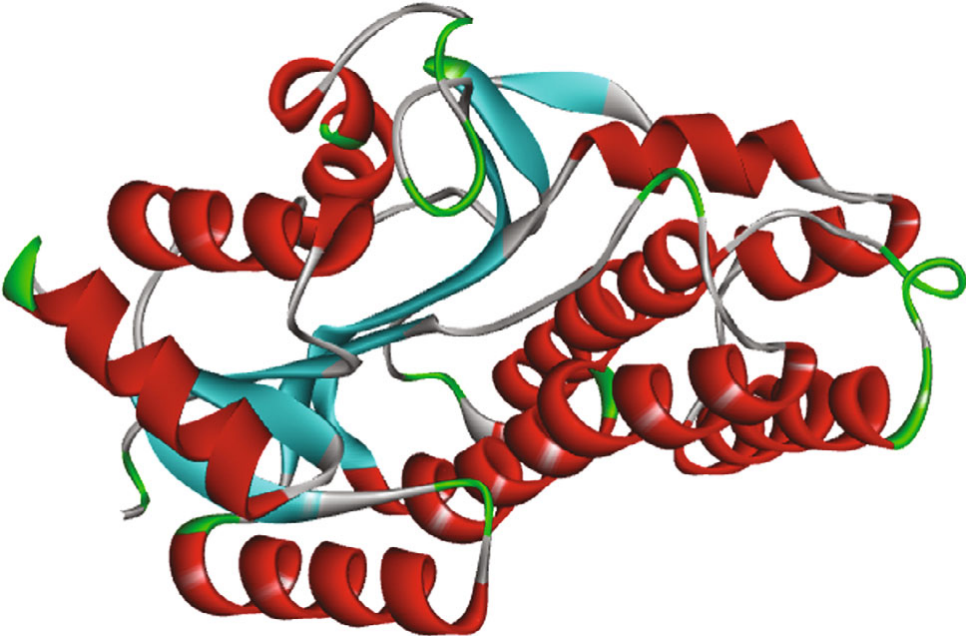
The 3D structure of selected target protein (PDB ID: 4Z3D).

The RCSB Protein Data Bank (https://www.rcsb.org/) provided the 3D crystal structure of human CBR‐1 protein (PDB ID: 4Z3D) determined by x‐ray diffraction. Figure 9 shows the protein′s 3D structure.

PyRx AutoDock software was used to evaluate binding energy and interaction with human CBR‐1 protein′s active areas to determine molecule‐target protein binding affinity. Table 8 lists all compounds with nearby binding scores and extensive intermolecular interactions.

**Table 8:** Binding affinity of ligands with targeted protein CBR‐1 (4Z3D).

**SL no.**	**Ligands**	**Binding affinity (kcal/mol)**	**Interacting amino acids**
1	Beta carotene (control)	−8.5	Ala235(A), Val230(A), Met234(A), Ile16(A), Val137(A), Pro227(A), Ile92(A), Phe94(A)
2	3,21Bis(heptafluorobutyriloxy)‐3,5,10‐pregnatrien‐20‐one	−8.7	Ala192(A), Ser190(A), Ser191(A), Met234(A), Gly228(A), Phe94(A), Gln105(A), Lys95(A), Ser139(A), Trp229(A), Met141(A), Val96(A), Tyr193(A)
3	Campesterol	−8.3	Pro130(A), Pro258(A), His5(A), Leu27(A), Leu255(A)
4	6‐Tert‐butyl‐2,3,4,9‐tetrahydro‐1H‐carbazol‐1‐one	−8.3	Gly91(A), Ala90(A), Asn89(A), Met234(A), Ile16(A), Pro227(A)
5	Stigmasterol	−8	Asn89(A), Gly17(A), Val108(A), Ile92(A), Phe94(A), Met234(A), Lys14(A)
6	Cholesta‐4,6‐dien‐3‐ol, (3.beta.)‐	−8	Leu17(A), Leu255(A), His5(A), Pro258(A)
7	Lupeol	−7.5	Leu27(A), Leu255(A), His5(A), Pro258(A)
8	Gamma.‐Sitosterol	−7	Leu27(A), Leu255(A), His5(A), Pro257(A), Pro258(A), Asp84(A)
9	Stigmastanol	−6.9	Leu27(A), Leu255(A), Pro257(A), Pro258(A), Pro130(A), Asp84(A)
10	4‐Campestene‐3‐one	−6.8	Leu27(A), Leu255(A), His5(A), Pro258(A), Phe28(A)
11	Benzeneethanamine, N‐trifluoroacetyl‐4‐hydroxy‐	−6.7	Asn89(A), Gly91(A), Ala90(A), Arg37(A), Leu61(A), Ile63(A)
12	Alpha.‐Tocospiro B	−6.7	Val251(A), Tyr252(A), Leu27(A), Leu255(A), Pro257(A), Pro258(A), Val85(A), His5(A), Phe28(A), Ser29(A)

The research employed beta carotene as a controlled medication with a binding affinity of −8.5 kcal/mol. The dimensions of the grid box were *X*, *Y*, and *Z* at 48.9284, 44.9892, and 58.2850 Å, respectively, with the grid center coordinates being *X* = −0.7507, *Y* = −0.2193, and *Z* = 0.3092.

**Figure 10:**
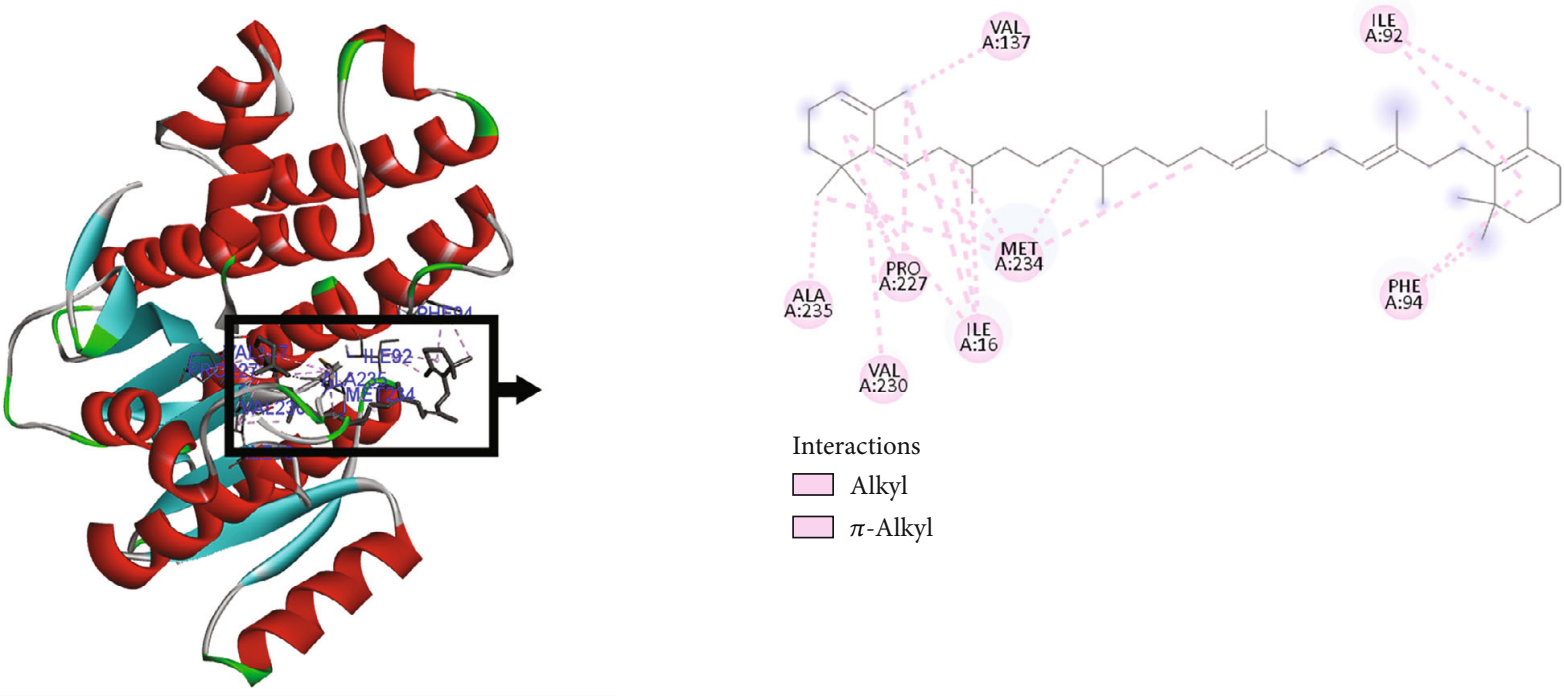
Protein–ligand complex (beta carotene and 4Z3D).

**Figure 11:**
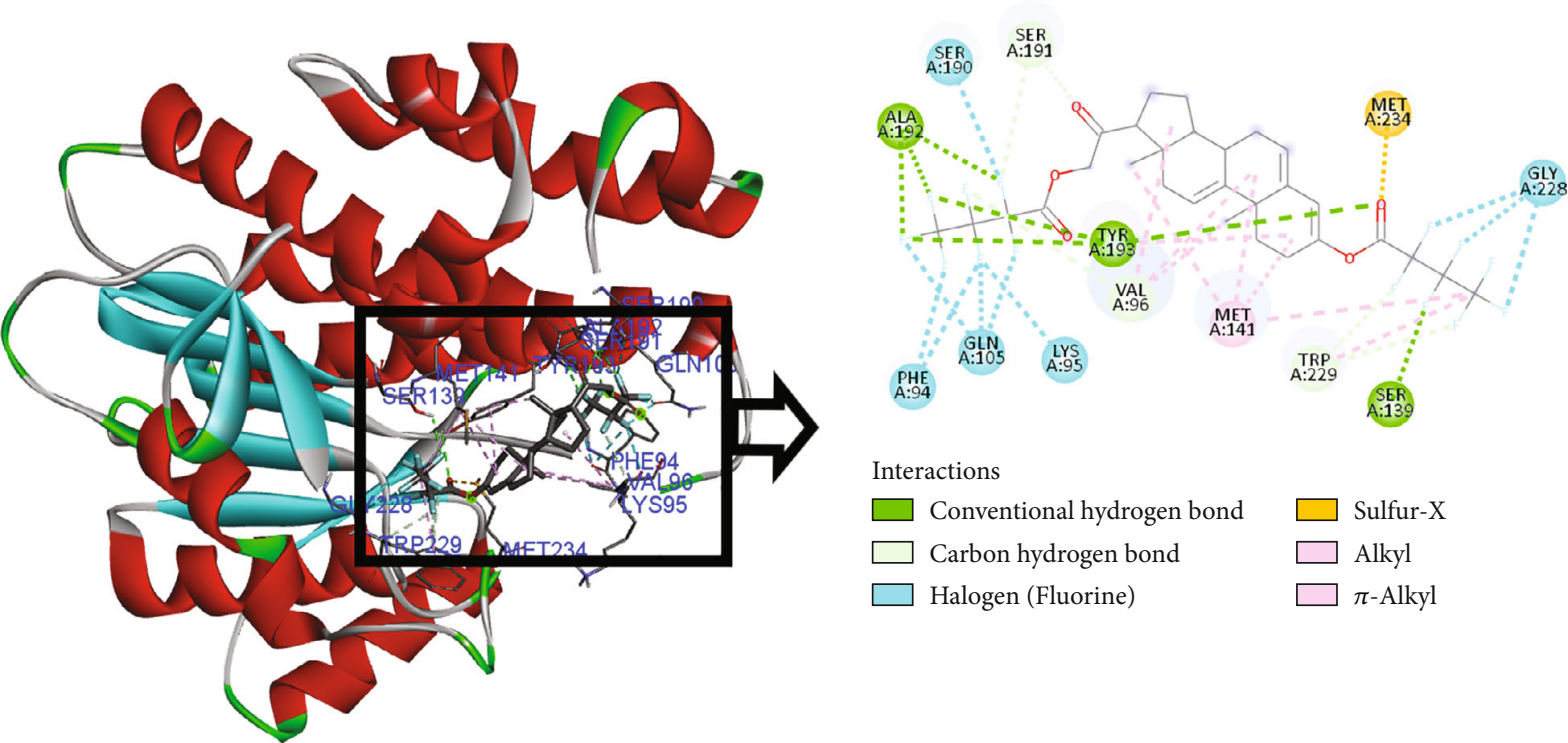
Protein–ligand complex (3,21bis(heptafluorobutyriloxy)‐3,5,10‐pregnatrien‐20‐one and 4Z3D).

**Figure 12:**
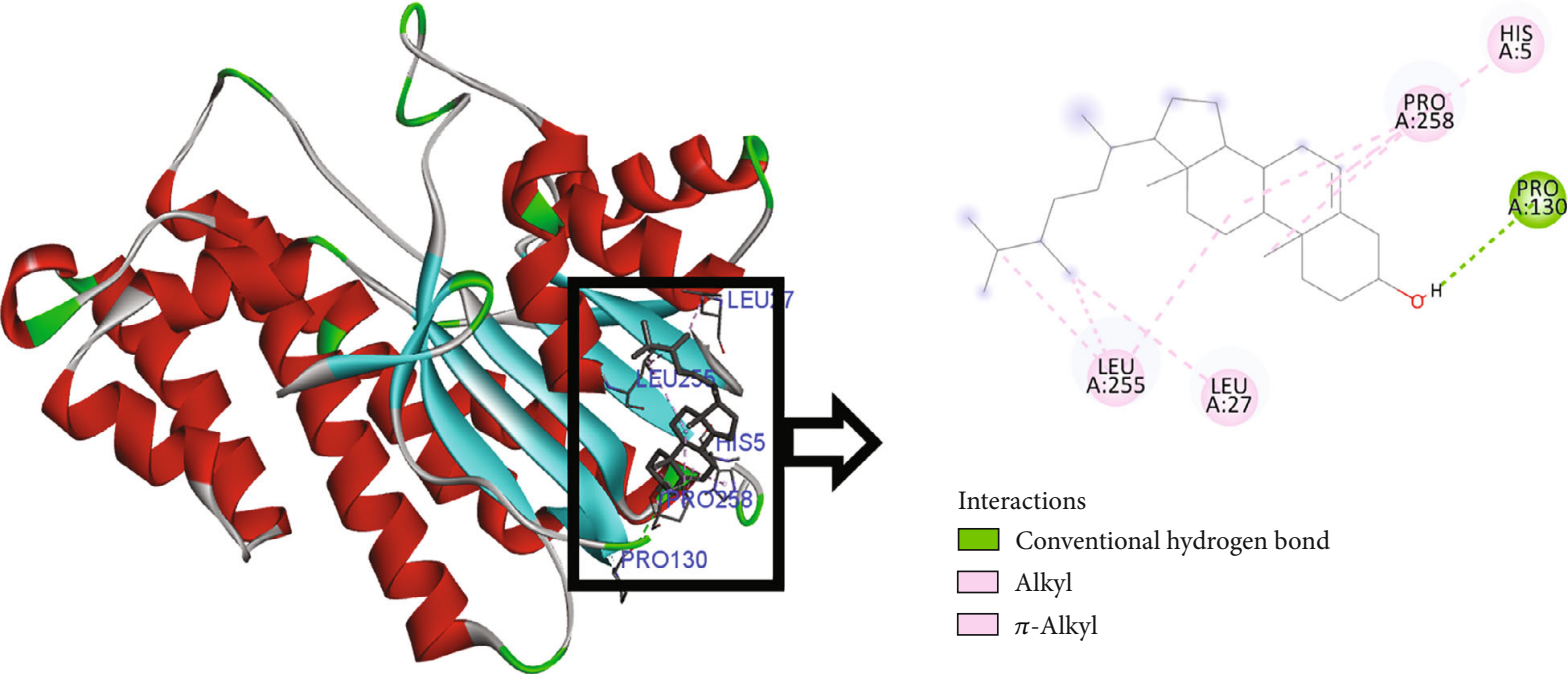
Protein–ligand complex (campesterol and 4Z3D).

**Figure 13:**
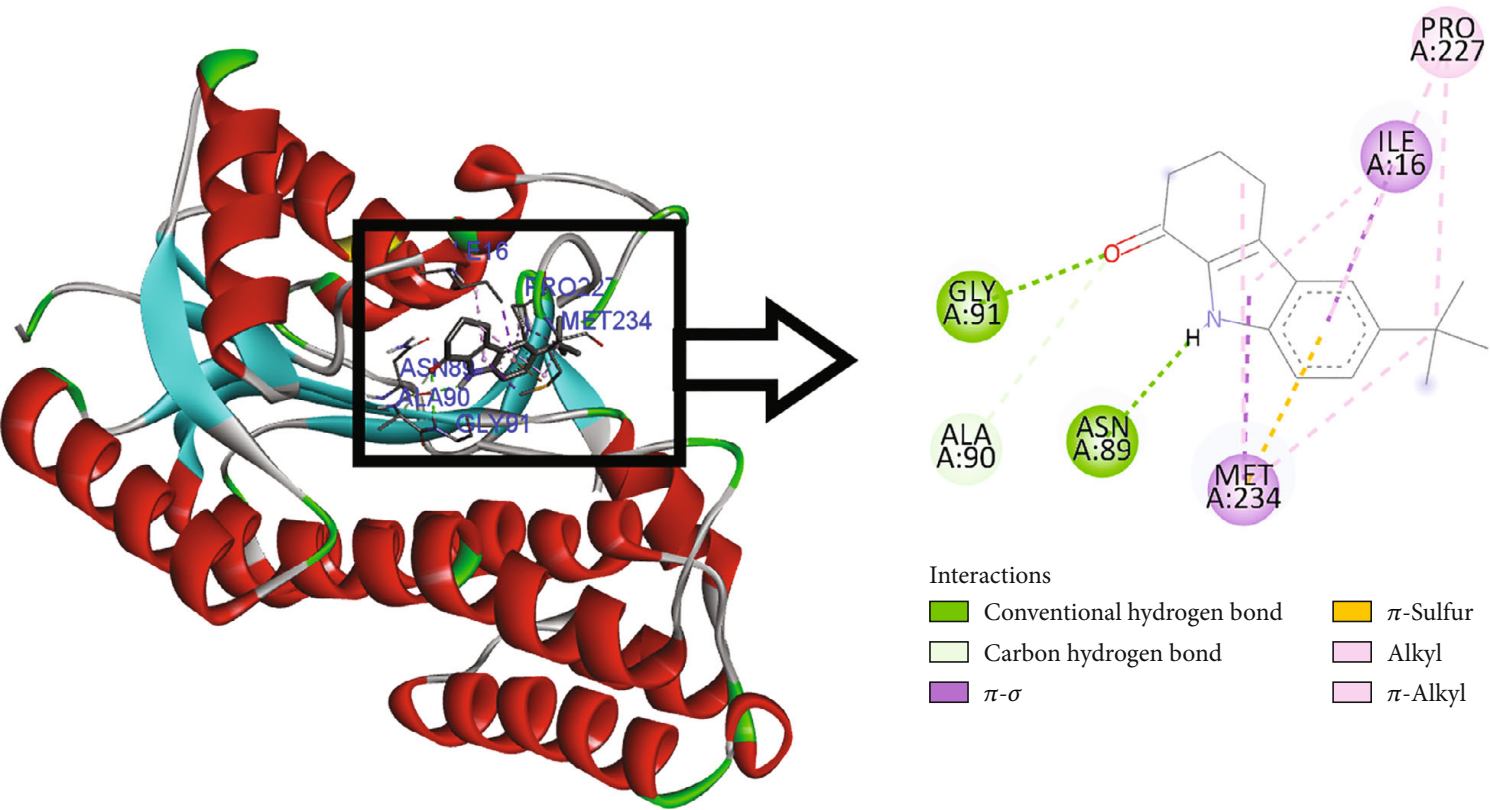
Protein–ligand complex (6‐tert‐butyl‐2,3,4,9‐tetrahydro‐1H‐carbazol‐1‐one and 4Z3D).

**Figure 14:**
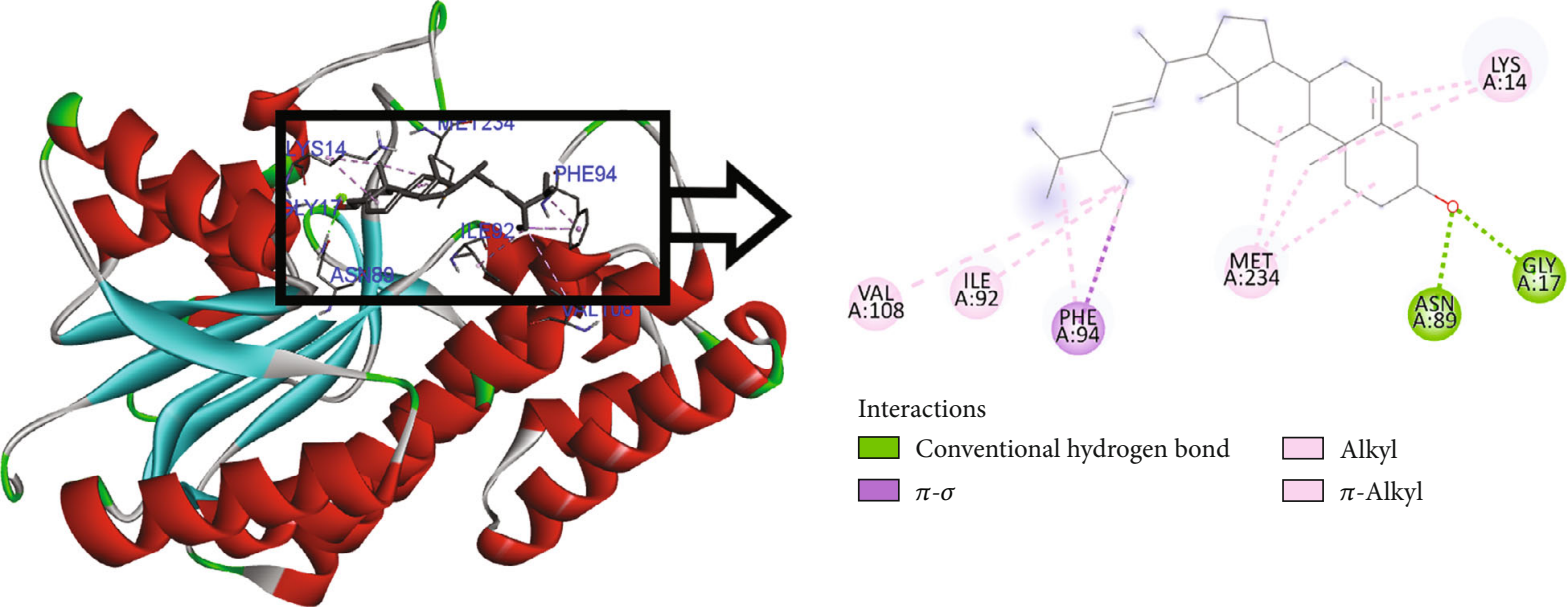
Protein–ligand complex (stigmasterol and 4Z3D).

**Figure 15:**
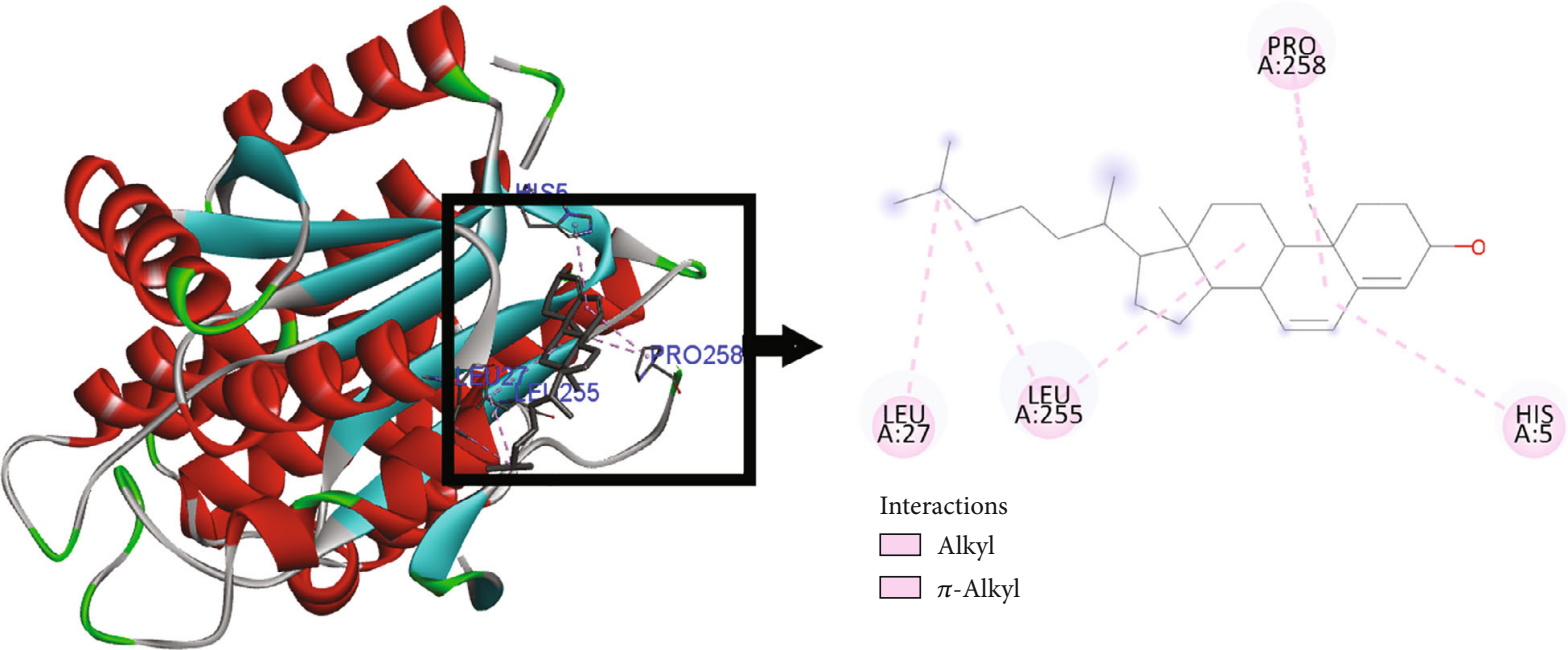
Protein–ligand complex (cholesta‐4,6‐dien‐3‐ol, (3.beta.) and 4Z3D).

The postdocking assessment revealed that the ligand 3,21bis(heptafluorobutyriloxy)‐3,5,10‐pregnatrien‐20‐one had the highest binding score of −8.7 kcal/mol. Four compounds, campesterol, 6‐tert‐butyl‐2,3,4,9‐tetrahydro‐1H‐carbazol‐1‐one, stigmasterol, and cholesta‐4,6‐dien‐3‐ol, (3.beta.), exhibited good binding affinity among all ligands, with binding affinity values of −8.3, −8.3, −8, and −8 kcal/mol, respectively. Ultimately, it can be said that these compounds exhibit advantageous binding, indicating their potential as antioxidant agents. Nonetheless, more experimental investigation is necessary to corroborate these results. The protein–ligand complexes interacting with these substances, in comparison to beta carotene (control), are shown in the accompanying pictures (Figures 10, 11, 12, 13, 14, and 15).

Data from the ADMET study suggested that alpha.‐tocospiro B emerges as the most promising chemical owing to its high absorption, poor water solubility, no BBB permeability, acceptable clearance, and lack of hepatotoxicity, carcinogenicity, and mutagenicity, though its potential for cytotoxicity needs further research. 6‐Tert‐butyl‐2,3,4,9‐tetrahydro‐1H‐carbazol‐1‐one and benzeneethanamine, N‐trifluoroacetyl‐4‐hydroxy also show a favorable profile, with high absorption and minimal toxicity concerns, making these two a feasible alternative for future investigation. In contrast, compounds such as 3,21‐bis(heptafluorobutyriloxy)‐3,5,10‐pregnatrien‐20‐one exhibit certain limitations, including low GI absorption and high MW, which might influence their therapeutic value. These findings provide a basis for prioritizing alpha.‐tocospiro B, 6‐tert‐butyl‐2,3,4,9‐tetrahydro‐1H‐carbazol‐1‐one, and benzeneethanamine, N‐trifluoroacetyl‐4‐hydroxy for subsequent in vitro and in vivo investigations to confirm their effectiveness and while optimizing their safety profiles (Table 9).

**Table 9:** ADMET analysis of some selected compounds found from GC‐MS analysis.

**Name**	**CID ID**	**ADME analysis**					**Pharmacokinetics**		**Druglikeness**	**Toxicity prediction**			
		**MW**	**HBA**	**HBD**	**Lipophilicity (iLOGP)**	**Water S.**	**GI absorption**	**BBB permeant**	**Lipinski violation**	**Hepatotoxicity**	**Carcinogenicity**	**Mutagenicity**	**Cytotoxicity**
3,21‐Bis(heptafluorobutyriloxy)‐3,5,10‐pregnatrien‐20‐one	550479	720.49	19	0	4.35	Poorly soluble	Low	No	2	Inactive	Active, 0.57	Inactive	Inactive
Campesterol	173183	400.68	1	1	4.97	Moderately soluble	Low	No	1	Inactive	Inactive	Inactive	Inactive
6‐Tert‐butyl‐2,3,4,9‐tetrahydro‐1H‐carbazol‐1‐one	685271	241.33	1	1	2.65	Moderately soluble	High	Yes	0	Active, 0.69	Inactive	Inactive	Inactive
Cholesta‐4,6‐dien‐3‐ol, (3.beta.)‐	14795191	384.64	1	1	4.77	Moderately soluble	Low	No	1	Inactive	Inactive	Inactive	Inactive
Stigmasterol	5280794	412.69	1	1	5.08	Moderately soluble	Low	No	1	Inactive	Inactive	Inactive	Inactive
Lupeol	259846	426.72	1	1	4.72	Poorly soluble	Low	No	1	Inactive	Inactive, 0.63	Inactive	Inactive
Gamma.‐Sitosterol	457801	414.71	1	1	5.07	Poorly soluble	Low	No	1	Inactive	Inactive	Inactive	Inactive
Stigmastanol	241572	416.72	1	1	5.17	Poorly soluble	Low	No	1	Inactive	Inactive	Inactive	Inactive
4‐Campestene‐3‐one	11988279	398.66	1	0	4.78	Poorly soluble	Low	No	1	Inactive	Inactive	Inactive	Inactive
Alpha.‐Tocospiro B	71452249	448.68	4	1	3.67	Poorly soluble	High	No	0	Inactive	Inactive, 0.56	Inactive	Active, 0.60
Benzeneethanamine, N‐trifluoroacetyl‐4‐hydroxy‐	572282	233.19	5	2	1.58	Soluble	High	Yes	0	Inactive	Inactive	Inactive	Inactive

## 4. Discussion

When compared with previous investigations on the pharmacological activities of medicinal plants, the present study on *R. leucophylla* demonstrates both strengths and limitations. For antioxidant activity, the MERL exhibited strong free radical scavenging capacity (IC_50_ = 28.87 * μ*g/mL), closely comparable to the synthetic standard BHT. This potency is consistent with findings from *Rondeletia odorata* fruit extract (IC_50_ value 25–30 *μ*g/mL) and higher than some other Rubiaceae members, highlighting the competitive antioxidant potential of *R. leucophylla* [34]. However, its TPC was moderate compared to highly phenolic‐rich plants such as *Camellia sinensis*, suggesting scope for fractionation to enrich active constituents. In terms of thrombolytic activity, *R. leucophylla* achieved 39.42% clot lysis, which is encouraging but lower than synthetic agents like SK (> 70%). Nonetheless, similar levels of clot lysis have been reported for other plant extracts, including *R. odorata*, supporting the idea that phytochemicals may provide natural fibrinolytic activity, albeit at moderate levels compared to standard drugs [35]. The antimicrobial profile of MERL was broad, inhibiting both Gram‐positive and Gram‐negative bacteria as well as fungi. This spectrum is consistent with reports on related genera such as *Coffea arabica*, which exhibit antibacterial activity attributed to chlorogenic acids and alkaloids [36]. However, the activity of MERL was moderate compared to purified plant‐derived compounds or standard antibiotics, highlighting the need for isolation and characterization of specific bioactive molecules.

The anti‐inflammatory, antidiarrheal, and hypoglycemic activities observed in MERL also align with earlier reports on Rubiaceae plants. For instance, *Morinda citrifolia* extracts have demonstrated significant anti‐inflammatory and antidiarrheal effects due to iridoids and flavonoids, while *Gardenia jasminoides* has shown hypoglycemic potential linked to its geniposide content [37, 38]. In our study, MERL produced responses comparable to standard drugs, which is a major advantage, suggesting therapeutic promise. Yet, the relatively higher doses required in comparison to synthetic drugs underscore the limitation of working with crude extracts rather than isolated compounds.

Recent studies have demonstrated that plant‐mediated nanoparticles exhibit enhanced therapeutic properties compared with crude extracts, including stronger antioxidant, antimicrobial, and anticancer effects, largely due to synergistic interactions between the metallic core and phytochemical capping agents [39–46]. For example, green‐synthesized silver and copper nanoparticles using medicinal plants such as *Ziziphora clinopodioides* and *Thymus fedtschenkoi* showed potent bioactivities and improved stability relative to conventional formulations [42], while other reports highlight superior cytotoxic and antimicrobial effects of biogenic nanoparticles across diverse plant systems [47–51]. In line with these findings, the rich phytochemical composition and broad pharmacological activities observed in *R. leucophylla* suggest that this species could serve as an effective reducing and stabilizing agent for the green synthesis of silver nanoparticles, offering ecofriendly nanomaterials with enhanced biomedical potential.

Overall, the advantage of this study lies in its integrative approach by combining in vitro, in vivo, and in silico methods which provide a broader pharmacological perspective than many earlier single‐method reports. The main disadvantage is that crude extracts cannot match the potency of pure synthetic drugs, and the absence of compound isolation restricts mechanistic clarity. Nevertheless, this work establishes *R. leucophylla* as a promising candidate for further bioactivity‐guided fractionation and drug discovery.

## 5. Limitations of the Study

Despite the promising findings, this investigation has certain limitations that warrant consideration. First, the pharmacological assessments were performed using crude MERL, without isolating or characterizing the individual bioactive constituents responsible for the observed activities. As a result, the precise mechanisms of action remain to be elucidated. Second, the in vivo experiments were restricted to rodent models, which, while informative, may not fully recapitulate the complexity of human pathophysiology. Consequently, extrapolation of the results to clinical settings should be approached with caution. Third, the in silico molecular docking and ADMET analyses, although useful for predicting druglikeness and interaction potential, provide only preliminary insights and require validation through rigorous pharmacokinetic and toxicological studies. Furthermore, the antimicrobial evaluation was limited to a relatively small panel of bacterial and fungal strains, leaving the broader spectrum of activity undetermined. Finally, the study did not investigate long‐term safety profiles, potential toxicity, or possible herb–drug interactions, which are essential considerations for the future therapeutic development of *R. leucophylla.*

## 6. Conclusion

This study presents the first integrated pharmacological evaluation of *R. leucophylla* through in vitro, in vivo, and in silico approaches. The methanolic extract exhibited notable antioxidant, thrombolytic, antimicrobial, anti‐inflammatory, antidiarrheal, and hypoglycemic activities, supported by phytochemical screening, GC‐MS profiling, and molecular docking. These findings validate its ethnomedicinal relevance and highlight its potential as a source of bioactive compounds. Future studies should focus on isolating active constituents, clarifying mechanisms of action, and assessing safety and pharmacokinetics. Moreover, considering its rich phytochemical profile and bioactivities, *R. leucophylla* may also serve as a promising candidate for the green synthesis of silver nanoparticles, offering ecofriendly nanomaterials with enhanced biomedical applications.

## Conflicts of Interest

The authors declare no conflicts of interest.

## Author Contributions

Najmus Sakib Minhaj: conceptualization, experimentation, investigation, formal analysis, and writing—original draft. Rajib Das: formal analysis, experimentation, investigation, writing—original draft, writing—review and editing, and visualization. Sadia Afreen Chowdhury: formal analysis, resources, writing—original draft, and editing. Monira Ahsan: resources, conceptualization, formal analysis, writing—review and editing, visualization, supervision, and project administration.

## Funding

No funding was received for this manuscript.

## Data Availability

All the data will be available upon request.
